# Endometrial mesenchymal stromal/stem cells improve regeneration of injured endometrium in mice

**DOI:** 10.1186/s40659-024-00484-3

**Published:** 2024-02-13

**Authors:** Tianqi Li, Rachel W.S. Chan, Raymond H.W. Li, Ernest H.Y. Ng, Songying Zhang, William S.B. Yeung

**Affiliations:** 1 Department of Obstetrics and Gynaecology, School of Clinical Medicine, Li Ka Shing Faculty of Medicine, The University of Hong Kong, Pokfulam, China; 2 Shenzhen Key Laboratory of Fertility Regulation, The University of Hong Kong Shenzhen Hospital, Pokfulam, China; 3 Centre for Translational Stem Cell Biology, The University of Hong Kong, Pokfulam, China; 4 Assisted Reproduction Unit, Department of Obstetrics and Gynaecology, Sir Run Run Hospital, School of Medicine, Zhejiang University, Zhejiang, China

**Keywords:** Endometrial stem cells, Endometrial regeneration, Fertility restoration

## Abstract

**Background:**

The monthly regeneration of human endometrial tissue is maintained by the presence of human endometrial mesenchymal stromal/stem cells (eMSC), a cell population co-expressing the perivascular markers CD140b and CD146. Endometrial regeneration is impaired in the presence of intrauterine adhesions, leading to infertility, recurrent pregnancy loss and placental abnormalities. Several types of somatic stem cells have been used to repair the damaged endometrium in animal models, reporting successful pregnancy. However, the ability of endometrial stem cells to repair the damaged endometrium remains unknown.

**Methods:**

Electrocoagulation was applied to the left uterine horn of NOD/SCID mice causing endometrial injury. Human eMSC or PBS was then injected into the left injured horn while the right normal horn served as controls. Mice were sacrificed at different timepoints (Day 3, 7 and 14) and the endometrial morphological changes as well as the degree of endometrial injury and repair were observed by histological staining. Gene expression of various inflammatory markers was assessed using qPCR. The functionality of the repaired endometrium was evaluated by fertility test.

**Results:**

Human eMSC successfully incorporated into the injured uterine horn, which displayed significant morphological restoration. Also, endometrium in the eMSC group showed better cell proliferation and glands formation than the PBS group. Although the number of blood vessels were similar between the two groups, gene expression of VEGF-α significantly increased in the eMSC group. Moreover, eMSC had a positive impact on the regeneration of both stromal and epithelial components of the mouse endometrium, indicated by significantly higher vimentin and CK19 protein expression. Reduced endometrial fibrosis and down-regulation of fibrosis markers were also observed in the eMSC group. The eMSC group had a significantly higher gene expression of anti-inflammatory factor *Il-10* and lower mRNA level of pro-inflammatory factors *Ifng* and *Il-2*, indicating the role of eMSC in regulation of inflammatory reactions. The eMSC group showed higher implantation sites than the PBS group, suggesting better endometrial receptivity with the presence of newly emerged endometrial lining.

**Conclusions:**

Our findings suggest eMSC improves regeneration of injured endometrium in mice.

**Supplementary Information:**

The online version contains supplementary material available at 10.1186/s40659-024-00484-3.

## Introduction

In recent years, stem cell-based therapy of damaged tissue and organ has become a hot topic in the regenerative field. Mesenchymal stem cells (MSC) have been regarded as the most promising cell source for tissue repair due to their extensive distribution and easy access [1]. Various types of MSC have been verified for their potential use in endometrial regeneration, from basic science to clinical studies [2–8]. However, they all have some shortcomings that limit further applications. Extraction of bone marrow-derived MSC and adipose-derived MSC requires an invasive procedure, increasing the difficulty of sample collection. While the easy extracted umbilical-cord derived MSC has potential threat of graft rejection, which also exists for the bone marrow-derived MSC. Although autologous transplantation of menstruation-derived stem cells alleviates immunogenicity, cell culture procedures might result in contamination due to the complex fluid composition including bacteria. Therefore, an alternate type of somatic stem cells with effective therapeutic properties and fewer side effects needs to be explored.

Endometrial stem/progenitor cells have proven to be responsible for the cyclical remodeling of human endometrium [9–11]. Using the surface markers CD140b (PDGRF-β) and CD146 identified stromal clonogenic cells in human endometrium [12]. The combination of these markers recognized human MSC-like pericytes from various tissues and organs [13] and revealed their perivascular location in the functional and basal layer of the endometrium. CD140b^+^CD146^+^ endometrial stromal cells have been proven to display high proliferative potential, multipotency and possess typical BM-MSC markers, hence they are referred as endometrial mesenchymal stem-like cells (eMSC). Gene profiling CD140b^+^CD146^+^ eMSC versus CD140b^+^CD146^−^ endometrial fibroblasts revealed eMSC are a distinct population of cells from endometrial fibroblasts that can differentially expressed several immunomodulatory genes [14, 15]. Together these findings suggest eMSC can interact with the immune system, exerting immunosuppressive functions through paracrine mechanisms and by direct contact with target cells. Since eMSC originate from a cyclically regenerating tissue, this cell population is an excellent candidate for tissue repair and regeneration. In particular endometrial regeneration deficiency can be caused by intrauterine adhesions (IUAs), which is commonly associated with infertility, recurrent miscarriage, and placental abnormalities. Asherman’s syndrome (AS) is a condition where IUAs form inside the uterus due to infection or trauma, thus reducing the area available for embryo implantation and result in infertility [16, 17]. We propose that eMSC will be a good cell source in repairing damaged endometrium because they are native cells of the endometrium for the cyclical regeneration. Here in this study, we investigated the functional potential of eMSC in the restoration of injured endometrium using an established mouse injury model.

## Materials and methods

### Isolation of endometrial cells from human tissues

Human endometrial tissue was obtained from 36 women aged between 41 and 54 years (mean age 47 years) with regular menstrual cycles at the time of recruitment and underwent total abdominal hysterectomy for benign pathologies (Supplementary Table S1). All recruited women signed the written consent form following counseling and did not take any exogenous hormonal therapy for at least three months before surgery. Ethical approval was obtained from the Institutional Review Board of The University of Hong Kong/Hospital Authority Hong Kong West Cluster (UW20-465).

Endometrial tissue was minced and digested with phosphate-buffered saline (PBS) containing 0.3 mg/ml collagenase III (Worthington Biochemical Corporation, Freehold, NJ, USA) and 40 µg/ml deoxyribonuclease type I (Worthington Biochemical Corporation) for 60 min at 37^o^C as described [18]. After two rounds of digestion, the dispersed cells were filtered through 40 μm sieves (BD Bioscience, San Jose, CA, USA). The red blood cells, cell debris and cell clumps were removed by density-gradient centrifugation using Ficoll-Paque (GE Healthcare, Uppsala, Sweden). Anti-CD45 antibody-coated Dynabeads (Invitrogen, Waltham, MA, USA) and anti-CD326 antibody-coated microbeads (Miltenyi Biotec Inc., San Diego, CA, USA) were used for removal of leukocytes and epithelial cells, respectively. Freshly purified stromal cells were seeded onto 10 cm dishes (BD Biosciences, San Jose, CA, USA) coated with fibronectin (1 mg/ml, Invitrogen) containing growth medium (GM) [DMEM/F-12 medium (Sigma-Aldrich, St Louis, MA, USA) with 1% L-glutamine (Invitrogen), 1% penicillin (Invitrogen) and 10% FBS (Invitrogen)]. Stromal cells were cultured until 80% confluency of the plate was reached in a humidified carbon dioxide incubator at 37^o^C. Cells were changed with growth medium every 7 days and were free of contamination.

### Magnetic selection of endometrial mesenchymal stem-like cells (eMSC)

To obtain eMSC (CD140b^+^CD146^+^ cells), two separate positive magnetic bead selections were conducted [19]. After in vitro expansion, stromal cells were incubated with PE-conjugated anti-CD140b antibody (R&D Systems, Minneapolis, MN, USA) for 45 min at 4^o^C and then with anti-mouse IgG1 coated magnetic microbeads (Miltenyi Biotec Inc.) for 15 min at 4^o^C. The CD140b^+^ population was acquired by applying the cell suspensions to MS columns (Miltenyi Biotec Inc.) in a magnetic field. The CD140b^+^ stromal cells were cultured in fibronectin-coated plates containing growth medium at 37^o^C in 5% CO_2_ for one week to allow degradation of microbeads during cell expansion. When the plates reached 80% confluent, the cells were trypsinized and incubated with anti-CD146 antibody-coated microbeads (Miltenyi Biotec Inc.) for 15 min at 4^o^C. CD140b^+^CD146^+^ eMSC were trapped in the column and flushed out for usage. The positive expression of CD140b and CD146 markers were confirmed by phenotypic study of eMSC [20]. Stromal cells at passage 1–4 were used in this study.

### Animal and housing condition

The mice were purchased and maintained in the Centre of Comparative Medicine Research at The University of Hong Kong. All experimental procedures performed in this study were approved by the Committee of Use of Live Animals in Teaching and Research, The University of Hong Kong. Housing of the mice followed standard laboratory conditions with a 12 h light/12 h dark cycle. Six mice were housed in one cage with free access to water and food.

### Mouse endometrial injury model

**Fig. 1:**
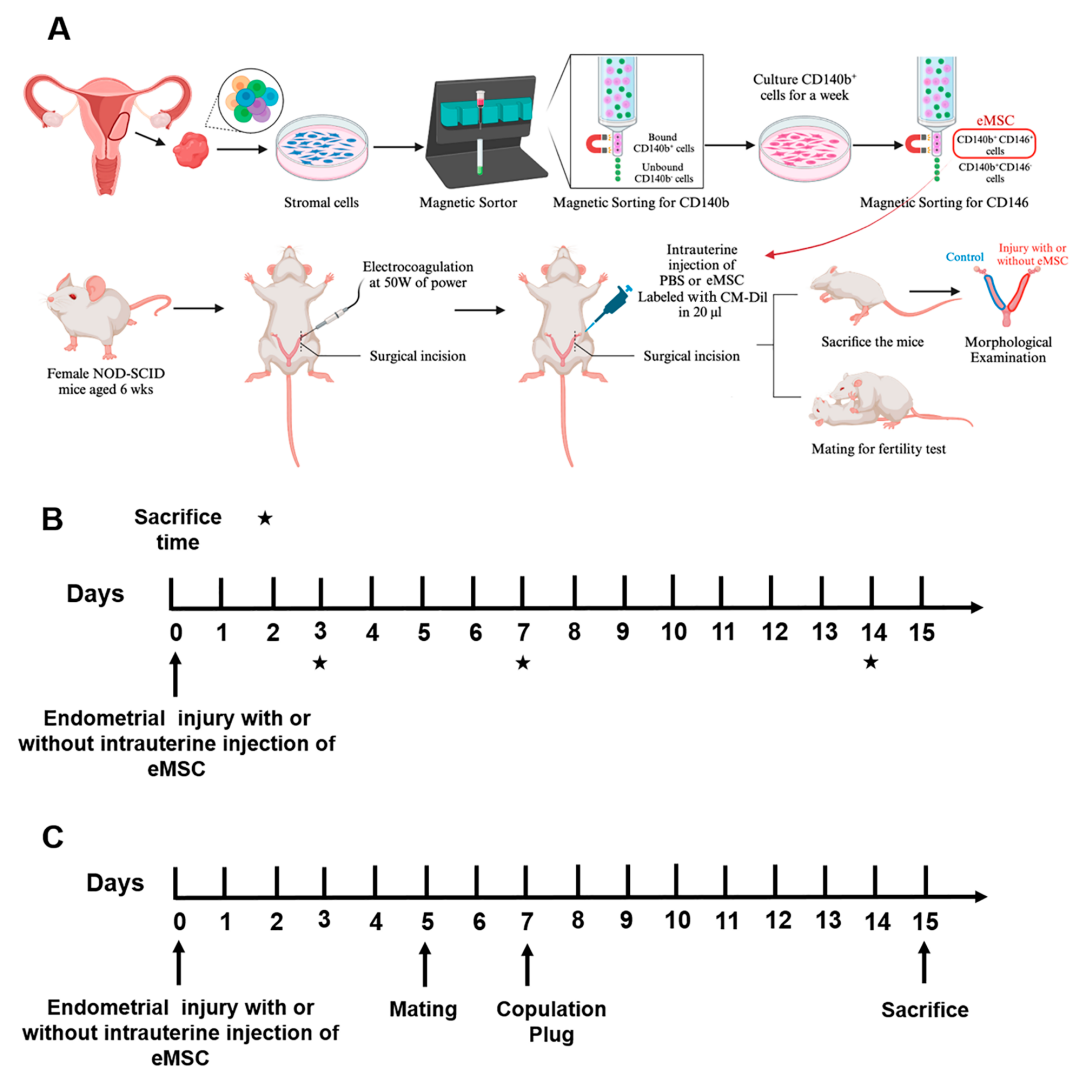
Experimental setup of the study. **(A)** A schematic diagram to illustrate the cell isolation, injury induction and eMSC intrauterine injection procedures. **(B)** Timeline of experimental methods. Endometrial injury was implemented by electrocoagulation on left side of the uterus of 6-week-old NOD-SCID mice at Day 0. Intrauterine injection of eMSC was performed on the same day and PBS was injected as untreated control. The mice were sacrificed on post-operative Day 3, 7 and 14 (black stars). **(C)** Timeline for fertility test, female mice would be mated with 8-week-old male mice on post-operative Day 5. Once copulation plug was observed, the female mice would be sacrificed on gestation Day 8. Abbreviation: eMSC, endometrial mesenchymal stem cells

Electrocoagulation was used to establish an endometrial injury model in 6-week-old NOD-SCID female mice as described previously [21]. Detailed experimental workflow is shown in Fig. 1A. Briefly, the mice at diestrus were operated after administration of ketamine (10 g/kg) and xylazine (80-100 mg/kg) by intraperitoneal injection. A vertical incision was made in the abdominal wall for exposure of the uterine horn (Supplementary Fig S1A) and a small incision was made in the upper region of the left uterine horn to insert the monopolar electrode into the lumen (Supplementary Fig S1B). Monopolar electrocoagulation was performed with 50 W power. While electrifying, the electrode pen was gradually moved out toward the incision of the uterine horn at constant pace. The whole process lasted for 3–4 s. After the uterine horn was returned into the abdominal cavity, abdominal wall and skin layer were sutured and disinfected. The right uterine horn of each mouse served as control.

A total of 48 NOD-SCID female mice were randomly allocated into one of two groups.

1. PBS group: 20 µl of PBS was injected into the injured uterine site immediately after the intrauterine injury .
2. eMSC group: 5 × 10^5^ eMSC labeled with CM-Dil dye resuspended into 20 µl of PBS were injected into the injured uterine site.

The mice were euthanized, and the uterine horns were collected at post-operative Day 3, 7 and 14 (*n* = 8/timepoint, Fig. 1B). A total of 8 uterine horns were assessed for each treatment/timepoint. Some of the harvested uterine horns were fixed in 4% paraformaldehyde overnight and processed into paraffin blocks (*n* = 4). Others were dissected, lysed for gene and protein examination (*n* = 4).

### CM-Dil labeled eMSC and in vivo tracing

After magnetic microbeads selection, the eMSC were incubated in PBS containing 2 mg/mL CM-Dil (Thermo Fisher Inc., Waltham, MA, USA) at 37^o^C for 5 min, followed by a 15-min incubation at 4^o^C. Labeling efficiency was examined under a fluorescence microscope and over 90% of the cell population was red-fluorescent cells (Supplementary Fig S1C). After intrauterine transplantation of CM-Dil labeled eMSC, uterine horns were harvested at post-operative Day 3, 7 and 14 and frozen in optimal cutting temperature (OCT) compound (Sakura Finetek, Torrance, CA, USA) in liquid nitrogen and stored at -80^o^ C until required. Frozen sectioned at 5 μm thickness were stained with dilution factor l in 1000 DAPI (Invitrogen) for 1 min to visualize the location and migration of eMSC in vivo.

### Masson trichrome staining

Paraffin sections (5 μm) were dewaxed and rehydrated before using the Masson’s Trichrome stain kit according to the manufacturer’s instruction (Beijing Solarbio Science & Technology Co., Ltd, Beijing, China). Images were captured using a Zeiss microscope (Carl Zeiss, Munich, Germany) and the TCapture software (Version 3.9, Tucsen Photonics, China). At least five fields of each transverse sections from a single uterine horn at each timepoint were analyzed with Image J software (US National Institutes of Health, USA). The blue positive area was divided by the total area of each field and the average value was calculated for each mouse section. The injured uterine horn was normalized against the control uterine horn from the same animal.

### Evaluation of endometrial morphology

Endometrial paraffin sections were routinely stained with hematoxylin and eosin (H&E) stain and examined under a Zeiss microscope (Carl Zeiss) and the images of were captured using TCapture software. Ten consecutive transverse sections from a single uterine horn at each timepoint were assessed. The average diameter of the endometrial cavity was calculated using the Image Pro Plus software (version 6.0; Media Cybernetics Inc, Rockville, USA).

### Immunohistochemical staining

After deparaffinization, rehydration and antigen retrieval procedures, the paraffin sections were incubated with 3% hydrogen peroxide (H_2_O_2_) for 10 min to quench the endogenous peroxidase. Blocked with 10% goat serum for 1 h, slides were then incubated with rabbit anti-cytokeratin 19 monoclonal antibody (1:200, Bioss Antibodies, Woburn, MA, USA) overnight at 4^o^C followed by biotinylated secondary antibody goat anti-rabbit IgG (1:200, Invitrogen) for 1 h. Slides were then covered with avidin-biotin complex (Vector Laboratories, Inc., CA, USA) for 30 min, visualized with diaminobenzidine (Dako, Santa Clara, CA, USA) and counterstained with hematoxylin for 30 s. Washing steps using PBS were conducted between each step and all incubations were conducted at room temperature unless otherwise specified. Sections were examined under a Zeiss microscope and the number of epithelial glands counted from the entire section of the endometrium using Image J software (US National Institutes of Health, USA).

### Immunofluorescent staining

The protocol for immunofluorescent staining was conducted as described above. Primary antibody rat anti-Ki67 monoclonal antibody (1:800, Abcam, Cambridge, UK) to detect cell proliferation and rabbit anti-CD31 polyclonal antibody (1:200, Abcam) to visualize blood vessels. Secondary antibodies goat anti-rat Alexa Fluor 488 (1:200, Invitrogen) and donkey anti-rabbit Alexa Fluor 568 (1:200, Invitrogen) were used for the conjugation of these primary antibodies, respectively. Cell nuclei were stained with DAPI (Invitrogen) for 1 min. Images were captured with a Nikon Eclipse Ti-S fluorescence microscope (Nikon, Melville, NY, USA) and the Image J software . Percentage of Ki67^+^ cells in the stromal compartment and number of blood vessels were counted using the ImageJ software (US National Institutes of Health, USA).

### Fertility test

To assess the endometrial function, fertility test was conducted to evaluate the receptivity of normal and injured uterine horns. Fertility evaluation were tested for all male mice prior to the experimental setup. Female mice without endometrial injury were mated with male mice to investigate the normal endometrial function. While for the experimental groups, female mice were randomly selected to mate with male NOD-SCID mice at post-operative Day 5 (Fig. 1C). Detection of a copulation plug was regarded as day 0.5 of gestation. Pregnant mice were euthanized day 8 of gestation and the number of implantation sites on each uterine horn were recorded.

### RNA extraction, reverse transcription and quantitative polymerase chain reaction (qPCR)

Total RNA was extracted from the uterine horns using Trizol (RNAiso Plus, Takara Bio Inc., Japan) according to the manufacturer’s instruction. RNA concentration was determined using Nanodrop 2000 spectrophotometer (Thermo Fisher Scientific) and balanced between both sides of uterus. Reverse transcription was accomplished using Prime Script RT reagent kit (Takara Bio Inc.) to convert from RNA to complementary DNA (cDNA). Quantitative PCR was then performed using Premix Ex Taq Master Mix (Takara Bio Inc.) with 7500 Real-Time PCR System (Applied Biosystems). Primers used for detection was listed in the supplementary table S2 and 18S was used as the internal control. All the experiments were performed in duplicates and the average value was calculated for comparison.

### Western blotting

Cell disruption buffer (Thermo Fisher Scientific) with proteinase inhibitor (Merck, Darmstadt, Germany) and phosphatase inhibitor (Merck) was used to lyse the uterine horns. After denatured at 95°C for 10 min, equal amounts of proteins were subjected to 10% SDS-PAGE and transferred to PVDF membranes. Membranes were blocked with 5% skimmed milk for 1 h at room temperature, followed by an overnight incubation with primary antibodies at appropriate concentrations (supplementary table S3) at 4^o^C. The membranes were incubated with corresponding HRP-conjugated secondary antibodies (listed in supplementary table S3) at room temperature for 1 h. Protein bands were then visualized using ECL detection reagents (Westsave UP™; AbFrontier, Seoul, Korea) and immediately exposed to x-ray film (Shenzhen Fumingwei Industrial Co., LTD, Shenzhen, China). Quantification of protein bands was achieved using ImageJ software and the expression of target proteins were calculated relative to internal control β-actin.

### Statistical analysis

Data were analyzed using the GraphPad PRISM software (version 8.0; GraphPad Software Inc., San Diego, CA, USA) and tested for normal distribution using the Shapiro-Wilk test. Two-tailed unpaired Student’s t-test for parametric data and Mann-Whitney test for non-parametric data were performed to determine the statistical significance between two groups. One-way ANOVA followed by Tukey’s test for parametric data and Kruskal-Wallis test followed by Dunn’s post-hoc test for non-parametric data were used for multiple group comparisons. Data are presented as mean ± standard deviation (SD). *P* < 0.05 was considered statistically significant.

## Results

### Human eMSC integrated and migrated to the injured uterine horn

**Fig. 2:**
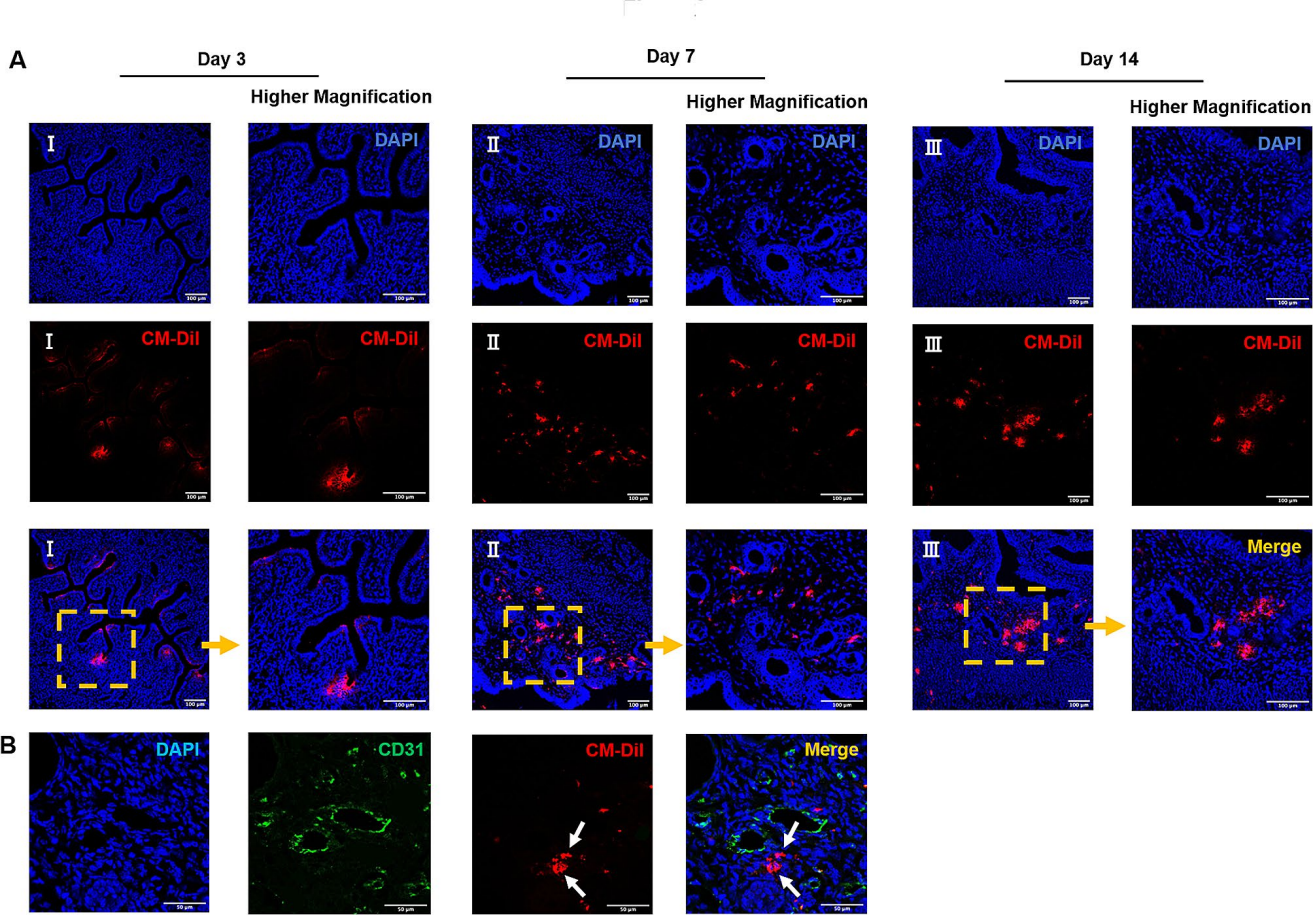
Localization of human eMSC. **(A)** Representative immunofluorescent images on the localization of human eMSC labeled with CM-DIL (red) in the injured mouse endometrium at post-operative Day 3 (I), 7 (II) and 14 (III). Scale bar: 100 μm. **(B)** Representative immunofluorescent images showing some eMSC (red) locate near the blood vessels (CD31+, green) at post-operative Day 14. Scale bar: 50 μm. Abbreviation: eMSC, endometrial mesenchymal stem cells

First, the extend and efficacy of injury in the left uterine horn was confirmed by histological examination on post-operative Day 3. In all traumatized mice, the histological disruption of the epithelial lining and stromal architecture was apparent (Supplementary Fig S1D). To evaluate the eMSC engraftment in the mouse injury model, frozen sections of the uterine horns at different timepoints were examined under the fluorescence microscope. Red fluorescent labeled eMSC were detected in all the mice from eMSC group across all the timepoints, indicating the integration of human eMSC into the mouse endometrium. On post-operative Day 3, the eMSC resided mainly beneath the luminal epithelium in the upper region of the endometrium (Fig. 2A I). Over time, the transplanted cells migrated further down into the endometrial stroma (Fig. 2A II) and by Day 14, some of the cells were detected near blood vessels (Fig. 2A III, B, white arrows), showing their homing tendency in areas with rich blood supply.

**Fig. 3:**
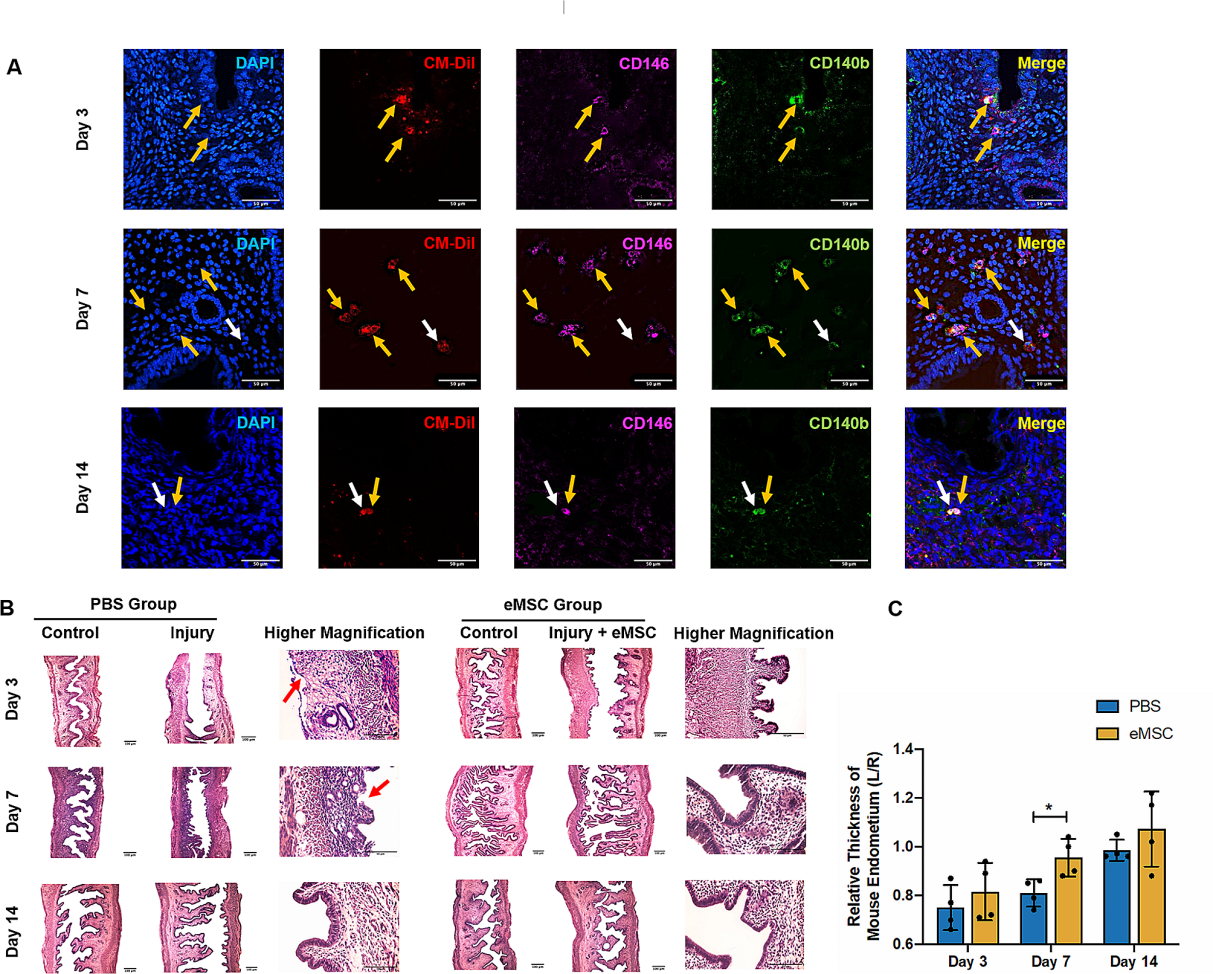
Expression of eMSC markers in transplanted cells and morphological features of the mouse endometrium. **(A)** Representative immunofluorescent staining images showing the expression of surface markers CD140b (green) and CD146 (pink) of transplanted human eMSC labeled with CM-Dil (red) at post-operative Day 3, 7 and 14. Scale bar: 50 μm. **(B)** Representative H&E images of mouse endometrium. Scale bar: 100 μm (lower magnification) and 50 μm (higher magnification). **(C)** Relative thickness of mouse endometrium in the PBS group (blue bars) and the eMSC group (yellow bars) (*n* = 4). The injured uterine horn (L) was normalized against the control uterine horn (R) from the same animal. Results are presented as mean ± SD; *P < 0.05. Abbreviation: eMSC, endometrial mesenchymal stem cells

To investigate whether the eMSC markers could be retained after transplantation, dual immunofluorescent staining for CD140b and CD146 was performed for all timepoints. As shown in Fig. 3A, some of the transplanted cells labeled with the CM-Dil dye co-expressed the eMSC surface markers (yellow arrow), while some cells no longer expressed these markers (white arrow). Although this finding demonstrated the presence of human eMSC in vivo, some of these cells may have differentiated after transplantation.

### Morphological integrity and thickness of mouse endometrium

Next, the morphological appearance of the normal and injured uterine horn in the PBS and eMSC groups were examined by H&E staining (Fig. 3B). The normal uterine horn displayed an intact endometrium with a complete luminal epithelium whereas loss of the upper region of the endometrium was observed in the injured horn at post-operative Day 3. Transplantation of eMSC ameliorated the damage of electrocoagulation and completeness of luminal epithelium was detected, indicating restoration of the injured mouse endometrium. By Day 14, the histological appearance of damaged endometrium was fully recovered (Fig. 3B).

The endometrial thickness was measured to assess the endometrial regeneration after injury. At post-operative Day 3, the endometrial thickness of the injured horn in the PBS and eMSC groups was similar (0.75 ± 0.09 fold and 0.82 ± 0.11 fold respectively, relative to the corresponding control horn; *n* = 4, Fig. 3C). Greater regeneration activity in the eMSC group was detected at post-operative Day 7 compared to the PBS group (0.96 ± 0.08 fold vs. 0.81 ± 0.06 fold respectively, *P* < 0.05, *n* = 4, Fig. 3C). Tissue repair continued in both groups and by Day 14, the endometrial thickness reached to 1.07 ± 0.15 fold for the eMSC group and 0.99 ± 0.04 fold for the PBS group (*P* = 0.43, *n* = 4).

### The proliferation activity in mouse endometrium

**Fig. 4:**
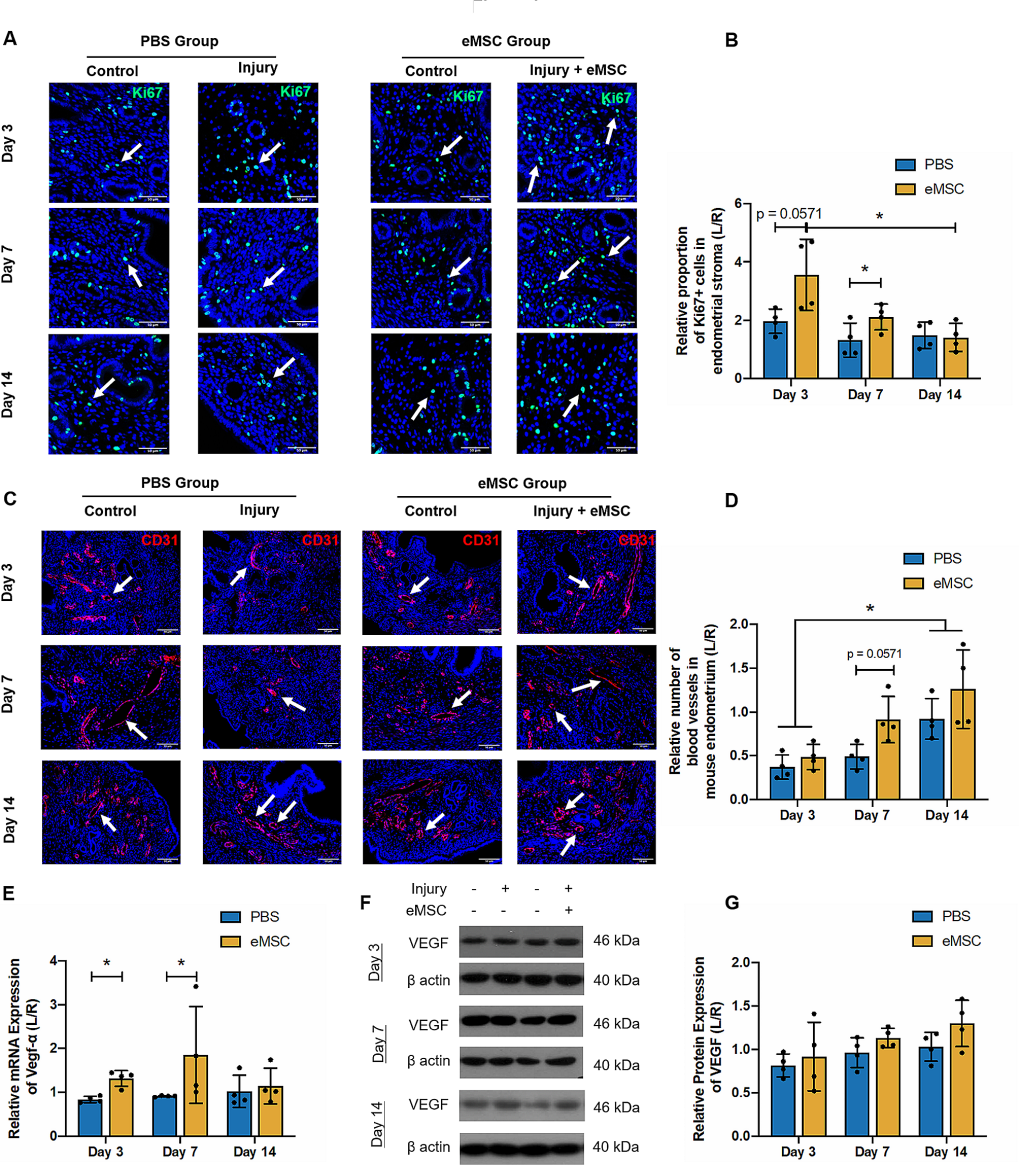
Stromal cell proliferation and angiogenesis in mouse endometrium. **(A)** Representative immunofluorescent images showing the nuclear expression of Ki67 protein (green) in mouse endometrium (white arrow) at different timepoints of post-transplantation. Scale bar: 50 μm. **(B)** Relative Ki67 protein expression in the PBS group (blue bars) and the eMSC group (yellow bars) (*n* = 4). **(C)** Immunofluorescent staining of blood vessels in the mouse endometrium with CD31 (red, white arrows). Scale bar: 50 μm. **(D)** Relative number of blood vessels in the PBS group (blue bars) and the eMSC group (yellow bars) (*n* = 4). **(E)** Relative gene expression of *Vegfa* in the PBS group (blue bars) and the eMSC group (yellow bars) on post-operative Day 3, 7 and 14 (*n* = 4). Representative western blotting images **(F)** and quantitative analysis **(G)** of VEGF protein expression in the PBS group (blue bars) and the eMSC group (yellow bars) normalized to β-actin (*n* = 4). Results are presented as mean ± SD; *P < 0.05. Abbreviation: eMSC, endometrial mesenchymal stem cells; Vegfa, vascular endothelial growth factor A

The proliferating activity in mouse endometrium was assessed by immunofluorescent staining of Ki67, which detects a nuclear antigen only in proliferating cells [22]. Figure 4A shows distribution of Ki67^+^ cells (green fluorescent signals, white arrows) from mouse origin in the uterine horns. The Ki67^+^ proliferating cells were detected at all the timepoints after injury. On post-operative Day 3, the eMSC group demonstrated a higher proliferative index than the PBS group but the difference did not reach statistical significance due to the large sample variation and small sample size (PBS group = 1.97 ± 0.42 fold vs. eMSC group = 3.56 ± 1.22 fold; *P* = 0.06; *n* = 4, Fig. 4B). By Day 7, significantly more Ki67^+^ cells were detected in the eMSC group compared to the PBS group. (PBS group = 1.01 ± 0.28 fold vs. eMSC group = 2.12 ± 0.44 fold; *P* < 0.05; *n* = 4, Fig. 4B). These findings suggested human eMSC may trigger mouse endometrial cells to turnover and replicate. Over time the proportion of proliferating cells gradually decreased. A 2.5 fold reduction could be observed in proliferating endometrial cells between Day 3 to Day 14 (*P* < 0.05).

### The structural components of the mouse endometrium

Apart from the increase in cell proliferation, restoration of endometrial structural components is also vital during the process of repair. Among all the structures, angiogenesis plays the most crucial role since nutrition and oxygen in blood supply are required for generation of new cells. In the present study, blood vessels in the uterine horns were visualized using CD31 immunofluorescent staining (Fig. 4C, white arrows). The number of blood vessels was relatively low three days after electrocoagulation. By Day 14, both groups displayed more blood vessels than Day 3 (Day 3: PBS group = 0.37 ± 0.14 fold vs. eMSC group = 0.49 ± 0.14 fold; Day 14: PBS group = 0.92 ± 0.23 fold vs. eMSC group = 1.01 ± 0.33 fold, *P* < 0.05, *n* = 4, Fig. 4D). The mRNA expression of vascular endothelial growth factor (Vegf)-α in endometrium with eMSC transplantation was higher at post-operative Day 3 (PBS group = 0.84 ± 0.07 fold vs. eMSC group = 1.31 ± 0.18 fold; *P* < 0.05, *n* = 4, Fig. 4E) and 7 (PBS group = 0.91 ± 0.02 fold vs. eMSC group = 1.85 ± 1.1 fold, *P* < 0.05, *n* = 4, Fig. 4E), suggesting the transplanted eMSC only promoted *Vegf-α* at gene level but no differences were detected at protein level (Fig. 4F, G). Whether the transplanted eMSC can promote formation of new vessels in the injured mice require further evaluation.

**Fig. 5:**
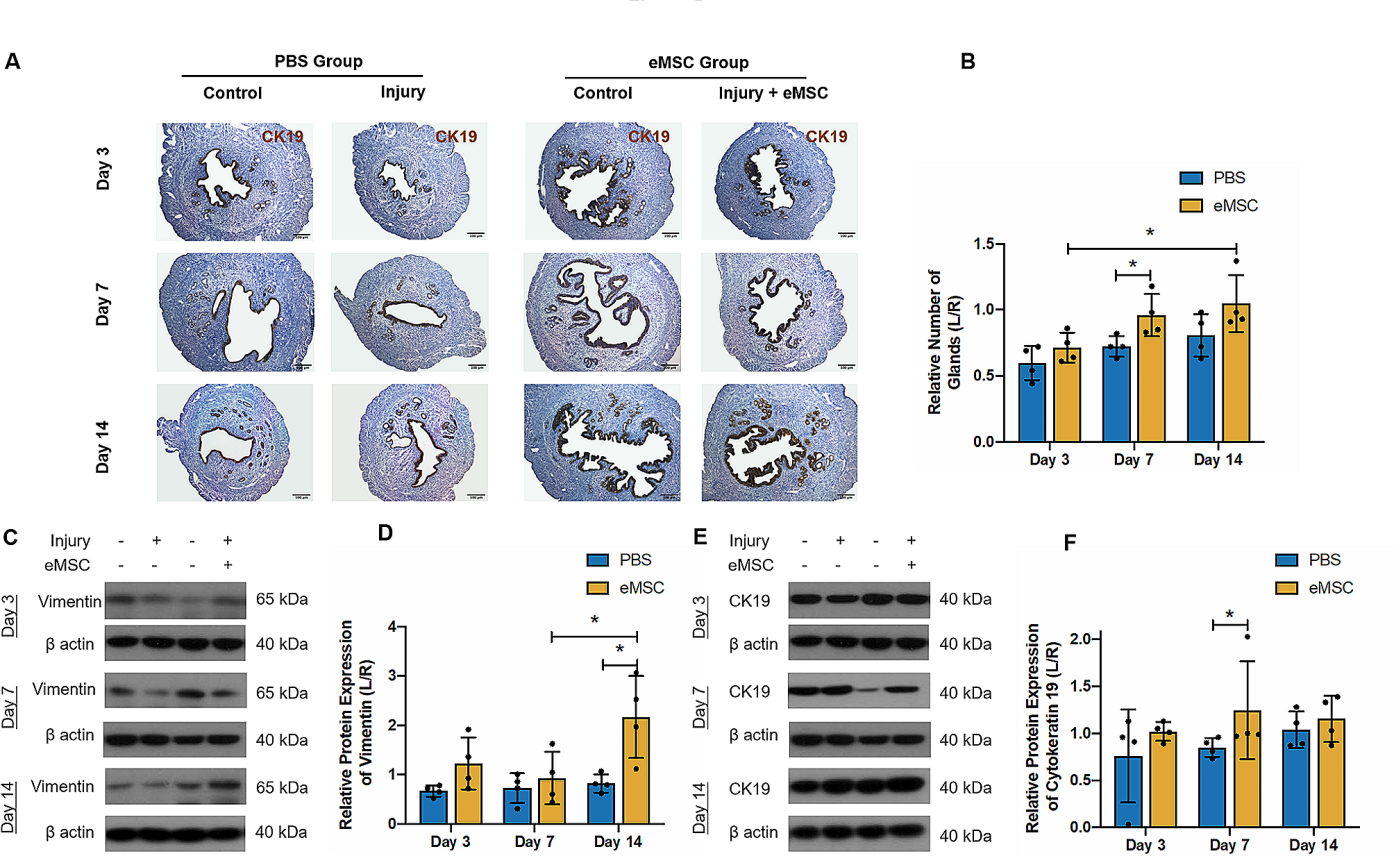
Effect of eMSC in restoring the endometrial epithelial and stromal compartment in the mouse endometrium. **(A)** Representative images showing the expression of cytokeratin 19 in mouse endometrium detected by immunochemistry staining. Scale bar: 100 μm. **(B)** Relative number of glands in the PBS group (blue bars) and the eMSC group (yellow bars) (*n* = 4). The injured uterine horn (L) was normalized against the control uterine horn (R) from the same animal. Representative western blotting images **(C)** and quantitative analysis **(D)** of vimentin protein expression in the PBS group (blue bars) and the eMSC group (yellow bars) normalized to β-actin. Representative western blotting images **(E)** and quantitative analysis **(F)** of cytokeratin 19 protein expression in the PBS group (blue bars) and the eMSC group (yellow bars) normalized to β-actin. Results are presented as mean ± SD, *P < 0.05; Abbreviation: eMSC, endometrial mesenchymal stem-like cells

Cytokeratin 19 (CK19), an epithelial cytoskeleton marker, recognizes the integrity of luminal epithelium and the proliferation of the glandular epithelial cells. Meanwhile vimentin is an intermediate filament protein abundantly observed in mesenchymal cells [23]. Their expression level indirectly reflects the extent of endometrial injury and repair in the uterine horns. Figure 5A demonstrated that on operative Day 3, almost half of the glands in the PBS group were lost (0.6 ± 0.13 fold, *n* = 4, Fig. 5B). By post-operative Day 7, more glands were formed in the transplantation group than the PBS group (PBS group: 0.72 ± 0.08 fold vs. eMSC group: 0.96 ± 0.16 fold; *P* < 0.05; *n* = 4, Fig. 5B). After 14 days of recovery, the number of glands in the transplanted group was comparable to the control horn (1.05 ± 0.22 fold, *n* = 4, Fig. 5B). Figure 5C and E show the representative images of vimentin and CK19 protein level. Relative low levels of vimentin and CK19 protein were present at post-operative Day 3 (vimentin: PBS group: 0.67 ± 0.11 fold vs. eMSC group: 1.23 ± 0.53 fold; *n* = 4, Fig. 5D; CK19: PBS group: 0.76 ± 0.49 fold vs. eMSC group: 1.02 ± 0.1 fold; *n* = 4, Fig. 5F). The effect of eMSC on epithelial cells production was detected on Day 7 (PBS group: 0.85 ± 0.1 fold vs. eMSC group: 1.25 ± 0.52 fold; *n* = 4, *P* < 0.05, Fig. 5F) and stromal regeneration occurred on Day 14 (PBS group: 0.82 ± 0.19 fold vs. eMSC group: 2.17 ± 0.83 fold; *n* = 4, *P* < 0.05, Fig. 5D). These results indicate that the transplantation of eMSC facilitated endometrial repair and improved the morphological integrity of the endometrium by enriching the number of glands, stroma and blood vessels.

### Fibrosis in mouse endometrium

**Fig. 6:**
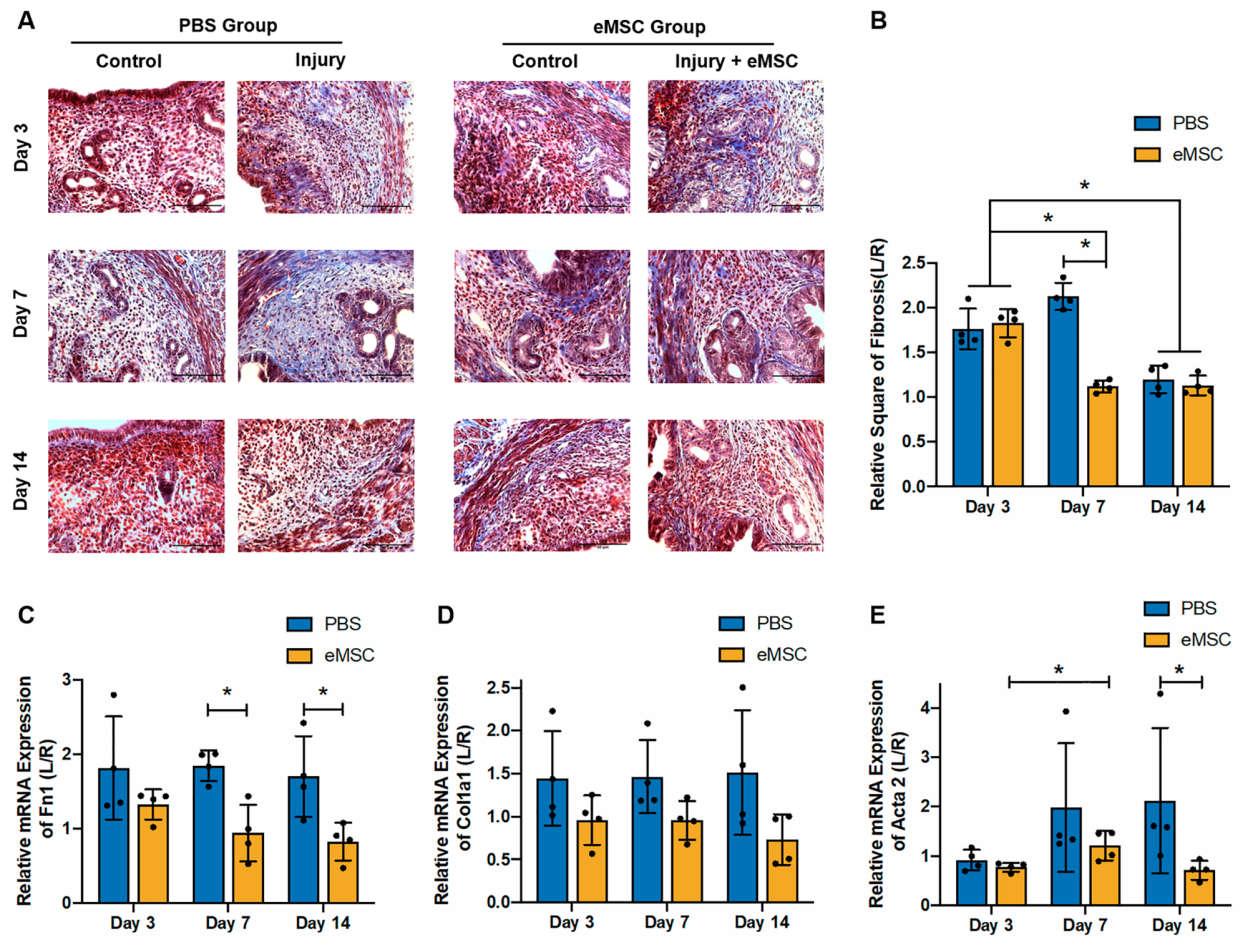
Effect of eMSC on endometrial fibrosis. **(A)** Representative Masson Trichrome stain images showing the degree of endometrial fibrosis in the mouse endometrium. Scale bar: 50 μm. **(B)** Relative square of fibrosis in the PBS group (blue bars) and the eMSC group (yellow bars) on Day 3, 7 and 14 post-surgery (*n* = 4). The injured uterine horn (L) was normalized against the control uterine horn (R) from the same animal. Relative gene expression of **(C)***Fn1*, **(D)***Col1A1* and **(E)***Acta2* in the PBS group (blue bars) and the eMSC group (yellow bars) at different post-operative timepoints (*n* = 4). Results are presented as mean ± SD; *P < 0.05. Abbreviation: eMSC, endometrial mesenchymal stem-like cells; Alpha 1; Acta, actin alpha; Col1a1, Collagen Type I; Fn1, Fibronectin

Fibrosis is a pathological wound repair process resulting from excessive replacement of the damaged tissue with connective tissue. The degree of collagen deposition in the injured uterine horns reflects the severity of tissue damage and the extent of repair. The Masson’s trichrome staining images showed that after endometrial injury, the presence of blue-stained collagen was evidently abundant (Fig. 6A). The eMSC group on Day 7 post-transplantation displayed significantly less area with positive stain than the PBS group (PBS group: 2.13 ± 0.15 fold vs. eMSC group: 1.12 ± 0.07 fold; *P* < 0.05; *n* = 4, Fig. 6B). By Day 14, the extent of fibrosis significantly declined compared to that in Day 3 (Day 3: PBS group: 1.77 ± 0.23 fold vs. eMSC group: 1.83 ± 0.16 fold; Day 14: PBS group: 1.2 ± 0.15 fold vs. eMSC group:1.13 ± 0.11 fold; *P* < 0.05; *n* = 4, Fig. 6B).

Furthermore, the mRNA expression of *Fn1* was significantly lower in the eMSC group at post-operative Day 7 and 14 than the PBS group (Day 7 PBS group: 1.85 ± 0.21 fold vs. eMSC group: 0.94 ± 0.38 fold; Day 14 PBS group: 1.7 ± 0.54 fold vs. eMSC group: 0.82 ± 0.26 fold; *P* < 0.05; *n* = 4, Fig. 6C). On the other hand, *Col1a1* expression remained unchanged (*n* = 4, Fig. 6D). The expression of *Acta2* in the eMSC group increased from Day 3 to Day 7 post-transplantation (Day3: 0.77 ± 0.1 fold; Day 7: 1.21 ± 0.3 fold; *P* < 0.05; *n* = 4, Fig. 6E). By post-operative Day 14, the transplantation of eMSC reduced the expression of this fibrotic gene when compared to the PBS group (PBS group: 2.12 ± 1.47 fold vs. eMSC group: 0.71 ± 0.19 fold; *P* < 0.05; *n* = 4, Fig. 6E).

### The inflammatory factors in mouse endometrium

**Fig. 7:**
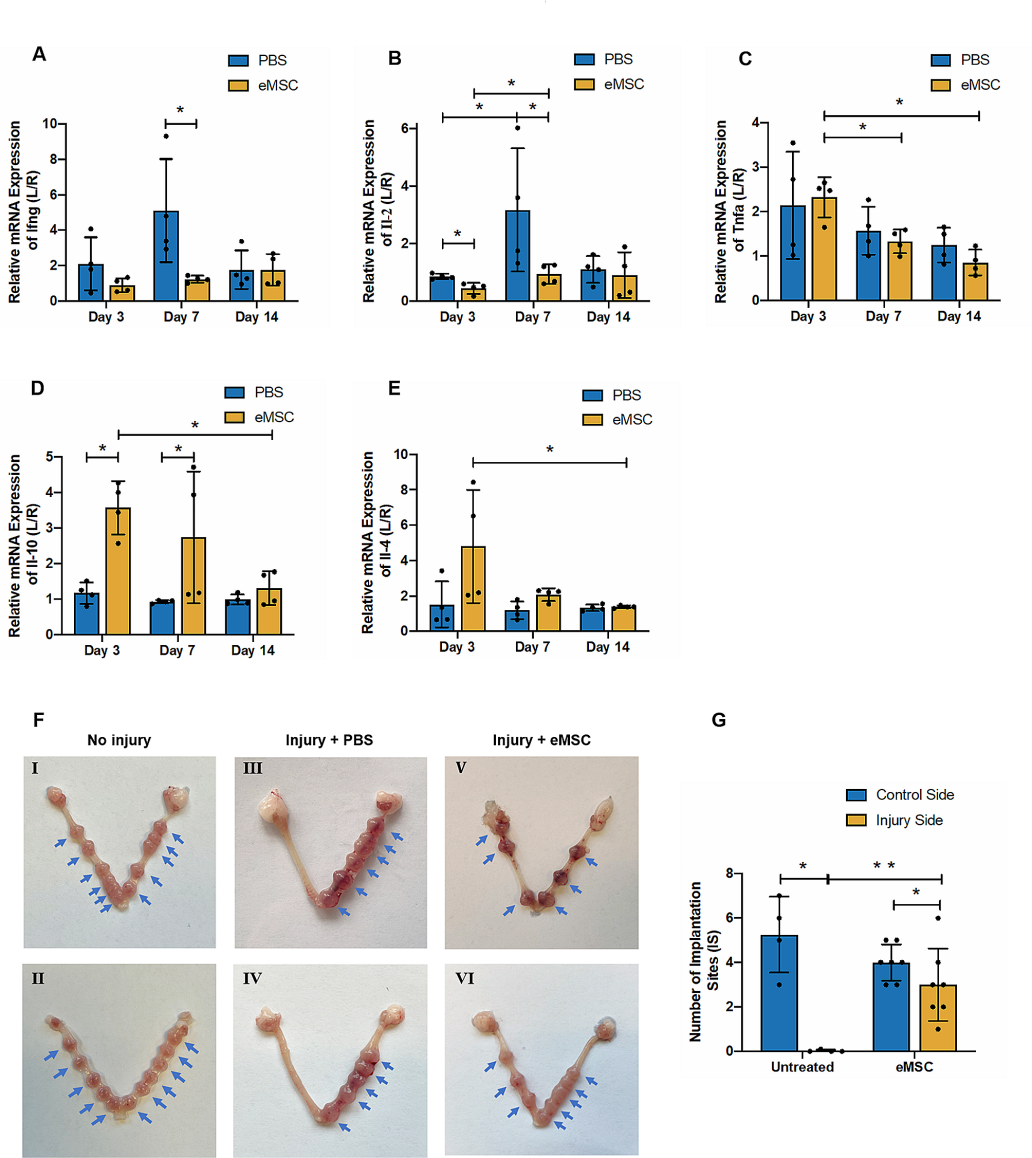
Effect of eMSC on inflammatory factors and the fertility restoration in the mouse endometrium. Relative gene expression of **(A)***Ifng*, **(B)***Il-2*, **(C)***Tnfa*, **(D)***Il-10* and **(E)***Il-4* in the PBS group (blue bars) and the eMSC group (yellow bars) at different post-operative timepoints (*n* = 4). The injured uterine horn (L) was normalized against the control uterine horn (R) from the same animal. **(F)** Images showing the uterine horns with the number of implantation sites (blue arrow) in (I and II) non-injury group, (III and IV) injured endometrium with PBS transplantation and (V and VI) injured endometrium with eMSC-transplantation. **(G)** Number of implantation sites on the control side (blue bars) and injured side (yellow bars) of the uterine horn (*n* = 4). Results are presented as mean ± SD, *P < 0.05. Abbreviations: eMSC, endometrial mesenchymal stem-like cells; Ifn, interferon; Il, interleukin; IS, implantation sites; Tnf, tumor necrosis factor

Inflammatory reactions are involved in the processes of tissue damage and repair [24]. Proinflammatory signals from dying cells activate immune cells, while anti-inflammatory factors are released during tissue repair. Previously, exosomes from UC-MSC have been proved to exhibit therapeutic potential on endometrial repair through macrophage immunomodulation [25]. In the present study, the production of pro-inflammatory factors such as interferon (Ifn)-γ, tumor necrosis factor (Tnf)-α and interleukin (Il)-2 at mRNA level in the mouse uterine environment between the PBS and eMSC groups was compared. The eMSC group exhibited reduced gene expression of *Ifng* and *Il-2* after 7 days of transplantation (*Ifng*: PBS group: 5.1 ± 2.9 fold vs. eMSC group:1.47 ± 0.31 fold; *P* < 0.05; *n* = 4, Fig. 7A; Il-2: PBS group: 3.17 ± 2.14 fold vs. eMSC group: 0.98 ± 0.34 fold; *P* < 0.05; *n* = 4, Fig. 7B). Although the gene expression of *Tnfa* was similar between the two groups, its level reduced over time in the transplantation group (Day3: 2.32 ± 0.46 fold; Day 7: 1.33 ± 0.27 fold; Day 14: 0.85 ± 0.29 fold; *n* = 4, *P* < 0.05, Fig. 7C). In contrast, the expression of anti-inflammatory factor Il-10 was significantly elevated in the eMSC group at Day 3 and 7 (Day3: PBS group: 1.17 ± 0.3 fold vs. eMSC group: 3.57 ± 0.75 fold; Day 7: PBS group: 0.93 ± 0.04 fold vs. eMSC group: 2.74 ± 1.86 fold; *P* < 0.05; *n* = 4, Fig. 7D). While the eMSC group tended to have higher Il-4 mRNA expression at Day 7 post-operation, but the difference did not reach statistical significance due to large sample variation and small sample size (*P* = 0.06; *n* = 4, Fig. 7E). These data suggest an anti-inflammatory profile at gene expression level was detected in the mouse endometrium after endometrial stem cell transplantation. However, this effect was not observed by Day 14. Whether eMSC has the ability to immunomodulate anti-inflammatory response require additional experiments.

### Fertility test

Since endometrium plays a key role during pregnancy, it was important to measure and compare the functionality of the regenerated endometrium after stem cell transplantation. The number of implantation site for each side the uterine horns was assessed. Figure 7F I and II showed both uterine horns of the non-injury group mice displayed successful embryo implantations and the number of fetuses were similar (blue arrows). In the PBS group, nearly no implantation site could be detected in the injured side (left) while the control side (right) remained unaffected (Fig. 7F III and IV, control side = 5.3 ± 1.71 vs. injured side = 0.2 ± 0.05; *P* < 0.05; *n* = 4, Fig. 7G). In the eMSC group, despite the number of implantation sites in the control side was still higher (Fig. 7F V and VI, control side = 4.0 ± 0.82 vs. injured side = 2.3 ± 1.1, *P* < 0.05; *n* = 7, Fig. 7G), the number increased dramatically in the eMSC transplantation side when compared to that of injured uterine horns. These data suggest transplantation of eMSC can repair the injured endometrium and partially restore the endometrial function for embryo implantation.

## Discussion

To date, numerous studies have demonstrated the therapeutic role of various types of somatic stem cells in tissue repair after endometrial injury, namely bone marrow-derived, adipose-derived, umbilical cord and menstruation-derived MSC [26]. The underlying mechanisms of this positive impact might be the secretion of growth factors or exosomes that stimulate angiogenesis and stromal proliferation, differentiation into complementary segments like endothelial cells, regulation of inflammatory reactions and inhibition of fibrotic endometrial regeneration [27–30].

Although these cells exhibit great regenerative potentials, native stem cells will be a better cell source to use for future clinical applications in treating women with intrauterine adhesions. In recent years, eMSC were identified in human endometrium which possess similar MSC features [31]. A set of eMSC which co-express the perivascular markers (CD140b^+^146^+^) express upregulated genes in relation to endometrial functions such as, inflammation, angiogenesis, vasoconstriction, cell communication and immunomodulation [14]. Base from our knowledge, this is the first study examining the functional potential of CD140^+^CD146^+^ cells in repairing injured endometrium using an in vivo mouse model.

The mechanism to induce damage in the endometrium varies among studies. Methods have been proven useful for the establishment of endometrial injury include ethanol perfusion, blade scrapping and electrocoagulation [21, 32–34]. The size of the uterine horns of NOD-SCID mice were too small to allow the endometrium to be scrapped. Since the incidence of intrauterine adhesion is usually the consequence of physical trauma to the endometrium in surgeries, we consider electrocoagulation would be a better tool for establishing the intrauterine injury model that mimics the pathological circumstances in the progression of IUA. Indeed, implementation of electrocoagulation successfully obliterated the luminal epithelium and part of endometrial tissue in the injured site, concurrently reducing the embryo implantation sites in the injured endometrium to a great extent, proving the effectiveness of our established model.

The CM-Dil labeling have been reported as a feasible method for in vivo tracing of transplanted cells in mouse tissue [35, 36]. This fluorescent dye exhibits great characteristics for cell tracing since it does not affect the survival, proliferation and differentiation of labeled cells and the fluorescence can be monitored for up to 30 days [37]. More importantly, there was no sign of label transfer between cells, indicating the stabilization of the fluorescent dye [38]. Our group utilized this cell tracer and demonstrated that eMSC can successfully merged into the injured sites of the mouse endometrium. As time went by, eMSC were randomly distributed in the uterine horn. EMSC congregated in the superficial layer of the endometrium in the initial days after transplantation. Gradually, these eMSC migrated towards the endometrial-myometrial junction and some of the cells resided in the perivascular regions - a proven physiological location for putative endometrial stem cells [39]. The homing capacity of different MSC populations has been well elucidated [40–42]. BM-MSC response to injury signals by leaving their residing niche through the blood vessels and entering the peripheral circulation to reach the injured site. The efficiency of MSC migration depends on their affinity to the injured tissue driven by mechanical or chemical factors [43]. Ischemia injury was demonstrated as the promotor of MSC migration to the endometrium [44]. In the present study, eMSC also exhibited migrating capacity after sensing the injury signals in mouse endometrium, which is similar to the responses of BM-MSC. Since eMSC are originated from endometrial tissue, it is possible that their affinity to the uterine microenvironment contributed towards the remarkable migration activity observed.

The occurrence of intrauterine adhesion result in endometrial fibrosis and scarring. The defected epithelial lumen and stroma are largely replaced by connective tissue, leading to the obstruction or even obliteration of the uterine cavity [30, 45]. A cell therapy to treat intrauterine adhesion requires the delivered cells to reconstruct a functional receptive uterine environment. Histological findings suggest the injured endometrium was morphologically restored after eMSC transplantation by post-operative Day 7. Increase in the endometrial thickness and the integrated epithelial lining, which is consistent with a previous study [21]. The uterine horn after eMSC transplantation also exhibited better structural reconstruction compared to the injury group, with more glandular and microvascular formation. The stimulation of angiogenesis displayed in the eMSC group might owe to the increase of *Vegf*. Similar observations in the mouse endometrium supports VEGF as a key angiogenic factor [46, 47]. Using the mouse menstrual-like model, the level of VEGF significantly elevated during endometrial breakdown then dropped remarkably after the endometrial tissue regenerated [46]. Decreased VEGF level in endometrium during the preimplantation stage may also have a negative impact on embryo placentation and development, leading to implantation failure [47]. The repair effect of eMSC on injured endometrium can be assessed by observing the content of endometrial stromal and epithelial cells, by the expression level of vimentin and cytokeratin 19, respectively. Endometrial repair begins at the luminal epithelium, followed by the proliferation of stromal cells that fulfill the aperture and enrich the endometrium in thickness [48]. In our study, protein level of CK19 significantly increased in the eMSC group after 7 days of recovery, indicating the effectiveness of eMSC on re-epithelization. Although proportion of Ki67^+^ stromal cells in the eMSC group was higher on Day 7, the facilitation of eMSC on the regeneration of stromal content did not appear until day 14 post-transplantation. These results fit well with the physiological changes in tissue regeneration processes.

Transplantation of eMSC also reduced the formation of fibrosis – a key pathological feature of intrauterine adhesion [49]. Gene expression of fibrotic markers *Fn1* and *Acta2* displayed a dramatic decrease after 7 and 14 days of recovery with transplanting eMSC, indicating a probable anti-fibrotic effect of these cells. In contrast, it was demonstrated that increasing fibrotic factors orchestrated by inflammatory factors can lead to the formation of endometriosis fibrosis [50–52]. Indeed, we detected an up-regulation of anti-inflammatory factor *Il-10* and down-regulation of pro-inflammatory factors *Ifng* and *Il-2* during the repair process, supporting the MSC function in fibrotic-relieving by secreting anti-inflammatory and anti-fibrotic factors [53].

Besides the endometrial architecture, the ultimate goal of intrauterine adhesion treatment is to restore fertility. Using eMSC as the cell-therapy tool, the fertility rate in the traumatized mice was significantly improved. T-helper type cytokines Ifn-γ and Il-2 were found incompatible with conception by mediating pregnancy loss, down-regulation of these pro-inflammatory factors and up-regulation of VEGF may have synergistically improved the endometrial receptivity in eMSC-transplanted uterine horns.

## Conclusion

Overall, this study demonstrated that electrocoagulation can be an effective tool for establishing the mouse endometrial injury model. Intrauterine injected eMSC displayed an integrating and migrating capacity in the mouse endometrium. These eMSC further presented an evident role on morphological repair of injured endometrium by promoting cell proliferation, angiogenesis, gland formation and the regeneration of epithelium and stroma, which overall improved the endometrium in thickness and integrity. From the functional aspect, eMSC transplantation partially restored fertility. Future studies on the molecular mechanisms and regulatory factors of in-vivo eMSC functions will help in better understanding the role of eMSC in uterine regeneration, offering a new alternative for cell-based therapy of the problematic IUAs.

### Electronic supplementary material

Below is the link to the electronic supplementary material.

**Supplementary Fig. 1** – Establishment and efficiency of endometrial injury. **(A)** Surgical exposure of the left side of mouse uterine horn. **(B)** Electrocoagulation of endometrium with an electrode for inducing endometrial injury. **(C)** Representative images showing the efficiency of eMSC (blue) with CM-Dil labeling (red). **(D)** Representative H&E images showing the morphology of control and injured mouse endometrium. Injured sites were indicated by the arrows.

**Supplementary Fig. 2** – uncropped scan of Western blots (Fig. 4F). The representative uncropped western blotting images is highlighted with red squares. 1: control side of PBS group; 2: injury side of PBS group; 3: control side of eMSC group; 4: eMSC transplantation side of eMSC group

**Supplementary Fig. 3** – uncropped scan of Western blots (Fig. 5C). The representative uncropped western blotting images is highlighted with red squares. 1: control side of PBS group; 2: injury side of PBS group; 3: control side of eMSC group; 4: eMSC transplantation side of eMSC group

**Supplementary Fig. 4** – uncropped scan of Western blots (Fig. 5E). The representative uncropped western blotting images is highlighted with red squares. 1: control side of PBS group; 2: injury side of PBS group; 3: control side of eMSC group; 4: eMSC transplantation side of eMSC group

Supplementary Material 5

Supplementary Material 6

## Data Availability

Not applicable.

## Declarations

### Completing interests

The authors declare that there are no completing interests.
