# Supplementary figures and images for: Endometrial mesenchymal stromal/stem cells improve regeneration of injured endometrium in mice
Source: Biol Res. 2024 Feb 13;57:6. doi: 10.1186/s40659-024-00484-3 (PMC10863157; doi:10.1186/s40659-024-00484-3)

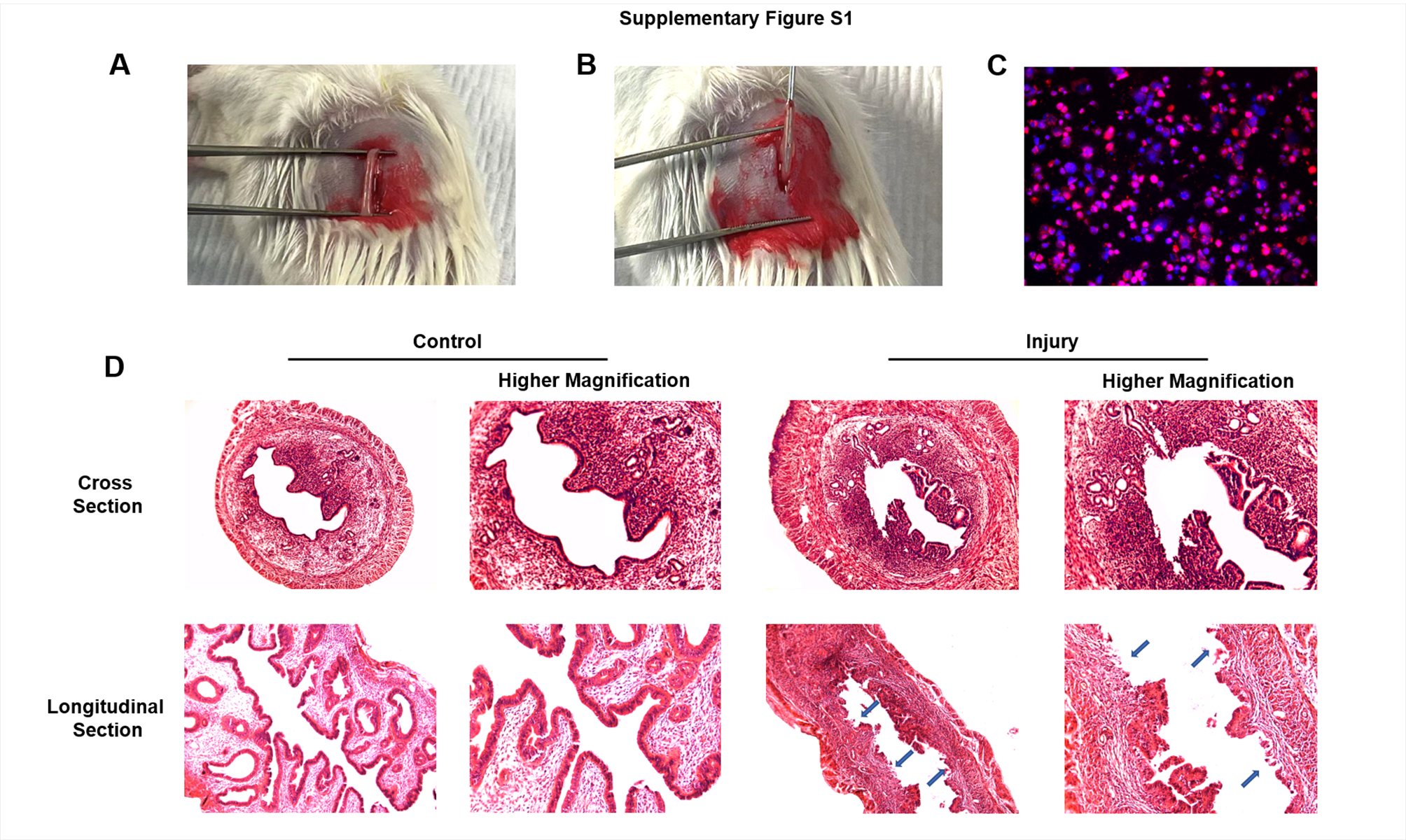

Supplement: Supplementary file 1 — Supplementary Fig. 1 – Establishment and efficiency of endometrial injury. (A) Surgical exposure of the left side of mouse uterine horn. (B) Electrocoagulation of endometrium with an electrode for inducing endometrial injury. (C) Representative images showing the efficiency of eMSC (blue) with CM-Dil labeling (red). (D) Representative H&E images showing the morphology of control and injured mouse endometrium. Injured sites were indicated by the arrows. [file 40659_2024_484_MOESM1_ESM.tif]

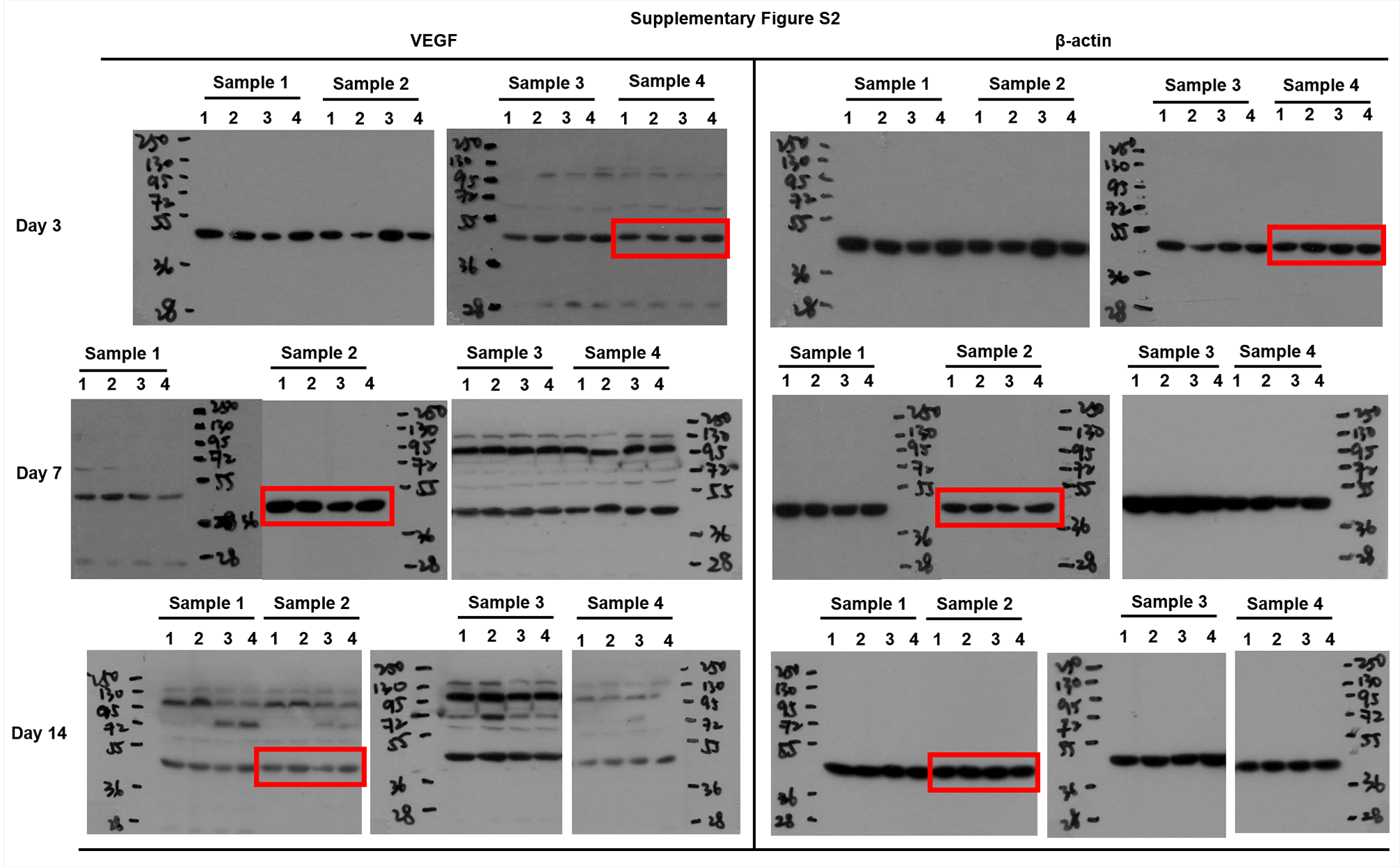

Supplement: Supplementary file 2 — Supplementary Fig. 2 – uncropped scan of Western blots (Fig. 4F). The representative uncropped western blotting images is highlighted with red squares. 1: control side of PBS group; 2: injury side of PBS group; 3: control side of eMSC group; 4: eMSC transplantation side of eMSC group [file 40659_2024_484_MOESM2_ESM.tif]

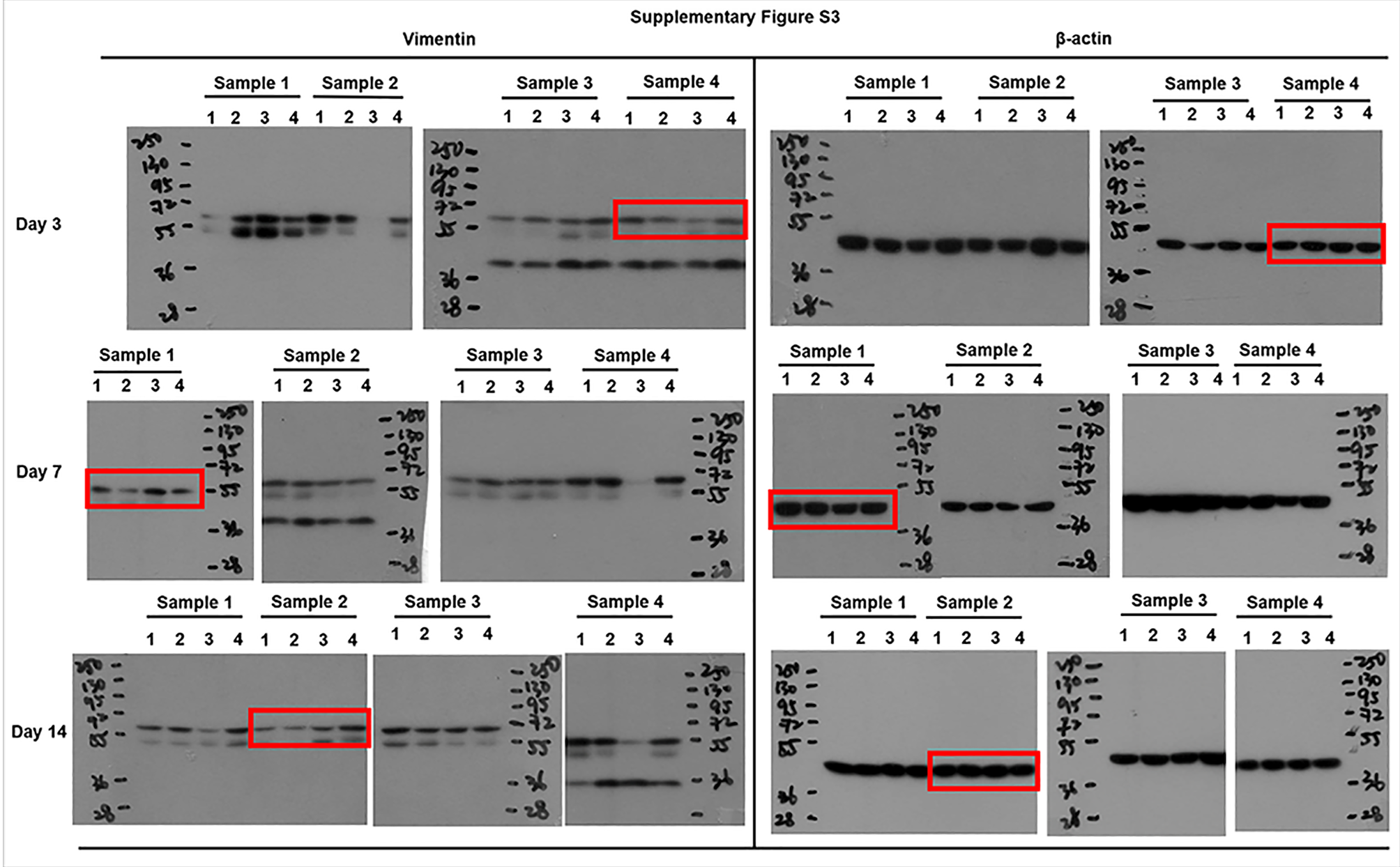

Supplement: Supplementary file 3 — Supplementary Fig. 3 – uncropped scan of Western blots (Fig. 5C). The representative uncropped western blotting images is highlighted with red squares. 1: control side of PBS group; 2: injury side of PBS group; 3: control side of eMSC group; 4: eMSC transplantation side of eMSC group [file 40659_2024_484_MOESM3_ESM.tif]

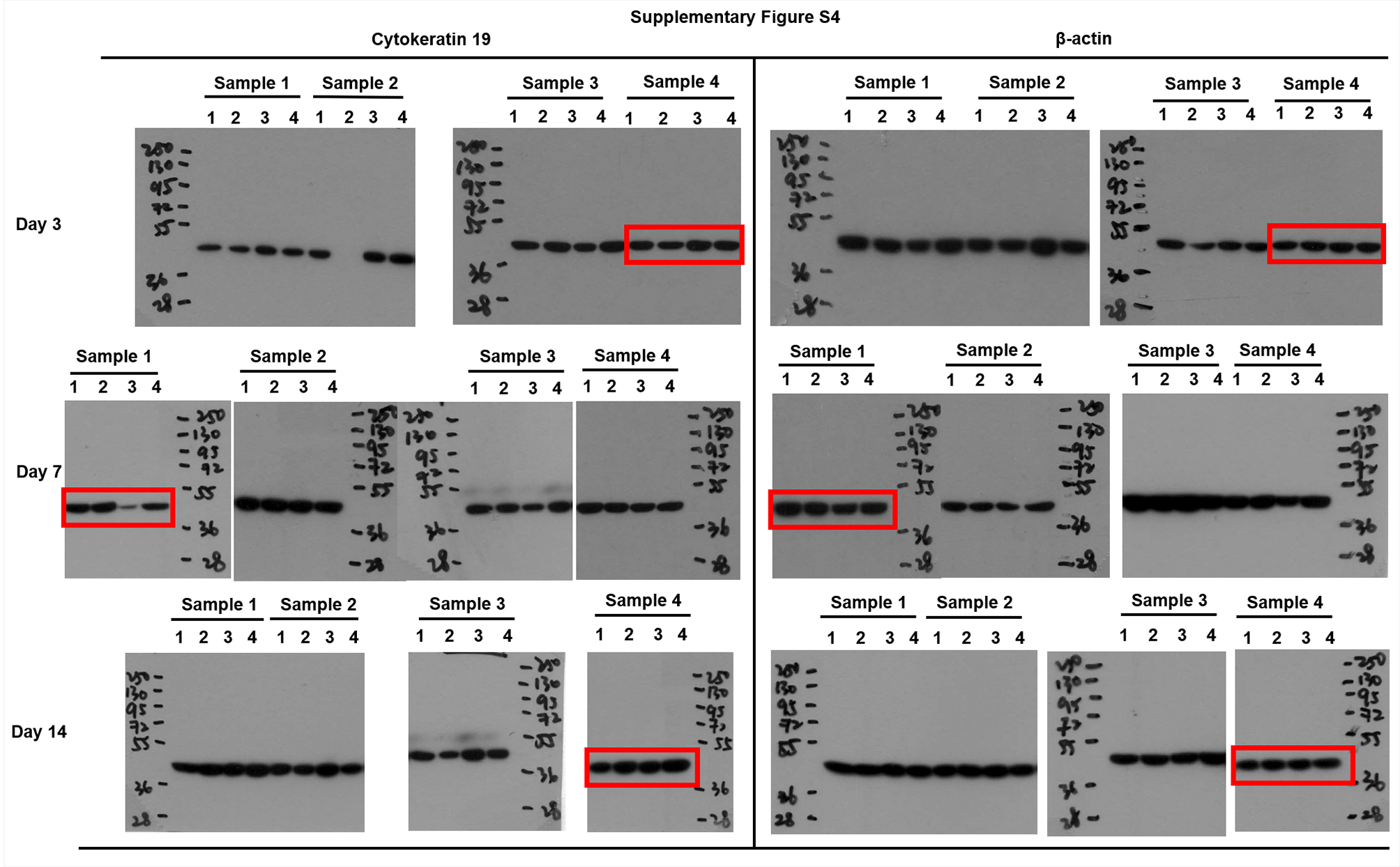

Supplement: Supplementary file 4 — Supplementary Fig. 4 – uncropped scan of Western blots (Fig. 5E). The representative uncropped western blotting images is highlighted with red squares. 1: control side of PBS group; 2: injury side of PBS group; 3: control side of eMSC group; 4: eMSC transplantation side of eMSC group [file 40659_2024_484_MOESM4_ESM.tif]
