# Supplementary material for: Endometrial mesenchymal stromal/stem cells improve regeneration of injured endometrium in mice
Source: Biol Res. 2024 Feb 13;57:6. doi: 10.1186/s40659-024-00484-3 (PMC10863157; doi:10.1186/s40659-024-00484-3)
Supplement: Supplementary file 5 — Supplementary Material 5 [file 40659_2024_484_MOESM5_ESM.docx]

Supplementary Table S1

| Patient No. | Age | Diagnosis |
| --- | --- | --- |
| 1 | 44 | Adenomyosis + Leiomyomas |
| 2 | 47 | Leiomyomas |
| 3 | 49 | Leiomyomas |
| 4 | 49 | Adenomyosis |
| 5 | 46 | Leiomyomas |
| 6 | 46 | Leiomyomas |
| 7 | 50 | Adenomyosis + Leiomyomas |
| 8 | 49 | Adenomyosis + Leiomyomas |
| 9 | 45 | Leiomyomas |
| 10 | 47 | Leiomyomas |
| 11 | 44 | Leiomyomas |
| 12 | 41 | Leiomyomas |
| 13 | 44 | Leiomyomas |
| 14 | 46 | Adenomyosis |
| 15 | 41 | CINIII HPV16+ |
| 16 | 52 | Leiomyomas |
| 17 | 54 | Leiomyomas |
| 18 | 43 | Adenomyosis |
| 19 | 46 | Leiomyomas |
| 20 | 46 | Adenomyosis |
| 21 | 48 | Leiomyomas |
| 22 | 48 | Adenomyosis |
| 23 | 47 | Leiomyomas |
| 24 | 47 | Adenomyosis + Leiomyomas |
| 25 | 47 | Leiomyomas |
| 26 | 50 | Leiomyomas |
| 27 | 44 | Leiomyomas |
| 28 | 47 | Leiomyomas |
| 29 | 50 | Leiomyomas |
| 30 | 45 | Leiomyomas |
| 31 | 44 | Adenomyosis + Leiomyomas |
| 32 | 48 | Leiomyomas |
| 33 | 51 | Leiomyomas |
| 34 | 47 | Leiomyomas |
| 35 | 48 | Leiomyomas |
| 36 | 43 | Leiomyomas |

Supplementary Table S2

| Gene name (Gene symbol) | Taqman Probes |
| --- | --- |
| Interleukin-2 (Il-2) | Mm00434256_m1 |
| Interleukin-4 (Il-4) | Mm00445259_m1 |
| Interleukin-10 (Il-10) | Mm01288386_m1 |
| Tumor necrosis factor (Tnf) | Mm00443258_m1 |
| Interferon gamma (Ifng) | Mm01168134_m1 |
| Vascular endothelial growth factor alpha (Vegfa) | Mm00437306_m1 |
| Fibronectin 1 (Fn1) | Mm01256744_m1 |
| Collagen type I alpha 1 chain (Col1a1) | Mm00801666_g1 |
| Actin alpha 2 (Acta2) | Mm00725412_s1 |

Supplementary Table S3

| Primary antibody | Dilution | Company | Catalog number |
| --- | --- | --- | --- |
| Goat Polyclonal Human/ Mouse/ Rat Vimentin | 2 μg/ml | R&D Systems | AF2105 |
| Rabbit Polyclonal VEGF | 1:1000 | Bioss | bs-0279R |
| Rabbit Monoclonal Cytokeratin 19 | 1:1000 | Bioss | bsm-52059R |
| Mouse Monoclonal β-actin | 1:2000 | Sigma Aldrich | 128018 |
|  |  |  |  |
| Secondary Antibody | | Dilution | Company |
| Anti-Mouse IgG | | 1:5000 | GE Healthcare |
| Anti-Rabbit IgG | |  |  |
| Anti-Goat IgG | |  |  |
